# Supplementary material for: A Cross-Sectional Study to Understand HPV Vaccine Hesitancy and Influencing Factors in Italian Adults
Source: Vaccines (Basel). 2025 May 31;13(6):599. doi: 10.3390/vaccines13060599 (PMC12197334; doi:10.3390/vaccines13060599)
Supplement: Supplementary file 1 [file vaccines-13-00599-s001.zip › vaccines-3619600-supplementary.pdf]

Supplementary Material

Table S1. HPV knowledge, attitudes and behaviours of respondents towards HPV infection and vaccination.

		N (%)
	**Positive**	**Negative**
How much do you believe that HPV vaccines are safe?	660 (36.2)	1161 (63.8)
How much do you believe that HPV vaccines are effective?	744 (40.9)	1077 (59.1)
	**Positive**	**Negative**
I believe that I am not particularly at risk of contracting HPV infection	236 (13.0)	1585 (87.0)
I believe that HPV infection does not cause serious illness	520 (28.6)	1301 (71.4)
	**Positive attitudes**	**Negative attitudes**
**Attitudes Variable**	343 (18.8)	1.478 (81.2)
	**Good**	**Limited**
HPV vaccination is useful in preventing cervical cancer	830 (45.6)	991 (54.4)
HPV vaccination is useful in preventing oral cancer (mouth)	552 (30.3)	1269 (69.7)
HPV vaccination is useful in males	551 (30.2)	1270 (69.8)
	**Good knowledge**	**Limited knowledge**
**Knowledge Variable**	638 (35.0)	1.183 (65.0)
**Intention to vaccinate Variable***	**High intention**	**Low/moderate intention**
	71 (26.4%)	198 (73.6%)

Dichotomized items and variables used in the models are reported.
*n=269, parents with children aged < 12 years

Table S2. Main barriers to HPV vaccination reported by respondents.

**Which of these statements more accurately explain the reason why you did not get vaccinated for HPV?** *(n=1,492)*				N (%)
	1° prioritiy	*2° prioritiy*	*3° prioritiy*	*Total*
The vaccination was not available at the time	669 (85.2)	87(11.1)	29 (3.7)	785
I am/was not aware of the possibility and/or my parents are/were not aware of the possibility	363 (67.6)	141(26.3)	33 (6.1)	537
I don't believe and/or my parents don't believe in the safety of the vaccines that are available as of yet	25 (22.7)	43 (39.1)	42 (38.2)	110
I don't believe and/or my parents don't believe in the effectiveness of the vaccines that are available as of yet	37 (28.7)	43 (33.3)	49 (38.0)	129
I don't trust and/or my parents don't trust sources that encourage HPV vaccination	38 (32.2)	40 (34.0)	40 (34.0)	118
I am aware of severe adverse reactions in family members/acquaintances after HPV vaccination	48 (27.8)	68 (38.0)	63 (35.2)	179
I don't believe and/or my parents don't believe in vaccinations and my opinion does not change for HPV vaccination	28 (34.6)	26 (32.1)	27 (33.3)	81
Vaccinations increase the risk of developing diseases like autism, multiple sclerosis and/or diabetes	67 (39.4)	58 (34.1)	45 (26.5)	170
Vaccinations increase allergic reactions	60 (32.6)	57 (30.0)	73 (38.3)	190
Difficulty in accessing free vaccination/Vaccination was too expensive for my cohort	74 (3.6)	73 (32.2)	80 (35.2)	227
Other (please specify)	83 (74.1)	10 (8.9)	19 (17.0)	112

For each statement, the selected preference percentages are reported.

Table S3. Reported reasons for HPV non-vaccination of all children.

**Which of the following statements best explains the reason why <u>your children</u> have not been vaccinated against HPV?** *max 3 items in ranking (n=760)*				
				**N (%)**
	1° prioritiy	*2° prioritiy*	*3° prioritiy*	**Total**
I am not aware of this possibility	262 (91.0)	21(7.3)	5 (1.7)	288
I have a male sex child	161 (70.9)	56 (24.7)	10 (4.4)	227
I don't believe in the safety of the vaccines that are available as of yet	35 (54.7)	20 (31.2)	9 (14.1)	64
I don't believe in the effectiveness of the vaccines that are available as of yet	29 (34.5)	38 (45.2)	17 (20.2)	84
I don't trust sources that encourage HPV vaccination	27 (34.6)	22 (28.2)	29 (37.2)	78
I am aware of severe adverse reactions in family members/acquaintances after HPV vaccination	16 (28.6)	20 (35.7)	20 (35.7)	56
I don't believe my child is at risk of contracting HPV	37 (44.6)	30 (36.1)	16 (19.2)	83
I don't believe in vaccinations and my opinion does not change for HPV vaccination	8 (25.0)	14 (43.8)	10 (31.3)	32
I would rather have my child vaccinated only for school required mandatory vaccinations	27 (45.6)	17 (29.3)	14 (24.1)	58
My child has already received too many vaccines	27 (38.0)	18 (25.3)	26 (36.1)	71
Vaccinations increase the risk of developing diseases like autism, multiple sclerosis and/or diabetes	9 (28.1)	10 (31.3)	13 (40.6)	32
Vaccinations increase allergic reactions	27 (39.1)	11 (15.9)	31 (44.3)	69
I had difficulty in accessing free vaccination	30 (47.6)	18 (28.6)	15 (23.8)	63
Other (please specify)	65 (89.0)	3 (4.1)	5 (6.8)	73

For each statement, the selected preference percentages are reported.

Table S4. Reported reasons for HPV non-vaccination of some children.

**Which of the following statements most accurately reflects the reason why <u>some of your children</u> have not received the HPV vaccine?** *max 3 items in ranking* **(***n=26)*				
				**N (%)**
	1° prioritiy	*2° prioritiy*	*3° prioritiy*	*Total*
I am not aware of this possibility	4 (66.7)	1 (16.7)	1 (16.7)	6
I have a male sex child	3 (60)	1 (20)	1 (20)	5
I don't believe in the safety of the vaccines that are available as of yet	1 (50)	0 (0)	1 (50)	2
I don't believe in the effectiveness of the vaccines that are available as of yet	2 (40)	2 (40)	1 (20)	5
I don't trust sources that encourage HPV vaccination	1 (100)	0 (0)	0 (0)	1
I am aware of severe adverse reactions in family members/acquaintances after HPV vaccination	2 (40)	2 (40)	1 (20)	5
I don't believe my child is at risk of contracting HPV	1 (33.3)	0 (0)	2 (66.7)	3
I don't believe in vaccinations and my opinion does not change for HPV vaccination	1 (100)	0 (0)	(0)	1
I would rather have my child vaccinated only for school required mandatory vaccinations	3 (60)	1 (20)	1 (20)	5
My child has already received too many vaccines	2 (50)	2 (50)	0 (0)	4
Vaccinations increase the risk of developing diseases like autism, multiple sclerosis and/or diabetes	0 (0)	0 (0)	1 (100)	1
Vaccinations increase allergic reactions	0 (0)	(0)	1 (100)	1
I had difficulty in accessing free vaccination	5 (83.3)	1 (16.7)	0 (0)	6
Other (please specify)	2 (66.7)	0 (0)	1 (33.3)	3

For each statement, the selected preference percentages are reported.

Table S5. Univariable analysis. Results are expressed as mean (SD) or frequency (%).

VARIABLES	NEGATIVE ATTITUDES N = 1,478	POSITIVE ATTITUDES N = 343	p-V [1, 2]
**Gender**			
Male	663 (45%)	169 (49%)	0.258
Female	812 (55%)	174 (51%)	
I prefer not to answer	3 (0%)	0 (0%)	
**Nationality**			
Italian	1,460 (99%)	337 (98%)	0.437
Non-Italian	18 (1%)	6 (2%)	
**Age**	54 (17.3)	54 (16.9)	0.427
**Geographical area**			
Northwest	401 (27%)	100 (29%)	0.576
Northeast	289 (20%)	61 (18%)	
Centre	296 (20%)	76 (22%)	
South/Islands	492 (33%)	106 (31%)	
**Marital status**			
Single	355 (24%)	76 (22%)	0.567
Married	792 (54%)	180 (52%)	
Separated/divorced	108 (7%)	26 (8%)	
Cohabiting	161 (11%)	48 (14%)	
Widowed	62 (4%)	13 (4%)	
**Educational level**			
University degree	365 (25%)	88 (26%)	0.925
Postgraduate	106 (7.2%)	25 (7.3%)	
High school or lower	1,007 (68%)	230 (67%)	
**Personal monthly income**			0.296
High (3,000 – over 6,000 euros)	92 (6%)	30 (9%)	
Medium (1,500 – 2,999 euros)	601 (41%)	130 (38%)	
Low (less than 1,499 euros)	632 (43%)	143 (42%)	
No income	153 (10%)	40 (12%)	
**With the financial resources available in your household, how do you make ends meet?**			
Definitely good/Fairly well	717 (49%)	181 (53%)	0.155
With some difficulty/ With many difficulties	761 (51%)	162 (47%)	
**Children**			
Yes	998 (68%)	227 (66%)	0.633
No	480 (32%)	116 (34%)	
**Occupation**			
Non-healthcare occupation	731 (49%)	142 (41%)	**0.002**
Healthcare worker	35 (2%)	20 (6%)	
Housewives	156 (11%)	39 (11%)	
Retired	426 (29%)	105 (31%)	
Students	27 (2%)	12 (4%)	
Unemployed	103 (7%)	25 (7%)	
**Specific Healthcare Occupation** *(n=55)*			
Medical Doctor	6 (17%)	7 (35%)	0.398
Nurse	11 (31%)	6 (30%)	
Obstetrician	6 (17%)	1 (5%)	
Healthcare Assistant	12 (34%)	6 (30%)	
**Chronic diseases**			
Yes	754 (51%)	187 (55%)	0.242
No	724 (49%)	156 (45%)	
**Cohabitants with disabilities**			
Yes	246 (17%)	61 (18%)	0.611

Table S5. Univariable analysis. Results are expressed as mean (SD) or frequency (%).

VARIABLES	NEGATIVE ATTITUDES N = 1,478	POSITIVE ATTITUDES N = 343	p-V [1, 2]
No	1,232 (83%)	282 (82%)	
**Self-Efficacy**			
"I'm capable of taking care of my health in an effective manner"	6.6 (1.6)	7.1 (1.6)	**<0.001**
"I'm a healthy person and I rarely suffer illnesses"	6.2 (2.1)	6.3 (2.4)	0.671
"I rarely seek help from healthcare services, and I handle my health problems on my own"	5.5 (2.4)	4.5 (2.9)	**<0.001**
**Health Literacy** (need for assistance in reading medical information)			
Low (never/rarely)	514 (35%)	66 (19%)	**<0.001**
High (sometimes/often/always)	964 (65%)	277 (81%)	
**Political orientation**			
Left	374 (25%)	91 (27%)	0.166
Moderate	477 (32%)	109 (32%)	
Right	335 (23%)	91 (27%)	
I prefer not to answer	292 (20%)	52 (15%)	
**Importance of religion** *(n=1467)*			
High	245 (21%)	71 (25%)	0.330
Medium	339 (29%)	78 (28%)	
Low	566 (48%)	129 (46%)	
I prefer not to answer	34 (3%)	5 (2%)	
**Religion**			
Catholic	1,079 (73%)	261 (76%)	0.659
Other religions	49 (3%)	9 (3%)	
None	294 (20%)	60 (17%)	
I prefer not to answer	56 (4%)	13 (4%)	
**Perceived quality of the Healthcare System**			
Good (high quality)	540 (36%)	168 (49%)	**<0.001**
Average (medium quality)	705 (48%)	135 (39%)	
Poor (low quality)	233 (16%)	40 (12%)	
**Have you ever received HPV vaccination?**			
Yes	81 (5%)	44 (13%)	**<0.001**
No	1,397 (95%)	299 (87%)	
**If children (12-18 yo), did you vaccinate your child for HPV?** *(n=234)*			
Yes	82 (42%)	30 (75%)	**<0.001**
No	112 (58%)	10 (25%)	
**If children (> 18 yo), did you vaccinate your child for HPV?** *(n= 839)*			
Yes	120 (18%)	57 (35%)	**<0.001**
No	558 (82%)	104 (65%)	
**If children 0-11 yo, from 0 to 100 how likely is it that you will vaccinate your children for HPV?** *(n=269)*	45 (36.6)	83 (29.8)	**<0.001**
**If No/ Not all my children have been vaccinated, from 0 to 100 how likely is it that you will vaccinate your children for HPV in the future?** *(n=786)*	25 (28)	35 (35)	**0.026**
**Knowledge**			
Limited	1,091 (74%)	92 (27%)	**<0.001**
Good	387 (26%)	251 (73%)	

Table S5. Univariable analysis. Results are expressed as mean (SD) or frequency (%).

VARIABLES	NEGATIVE ATTITUDES N = 1,478	POSITIVE ATTITUDES N = 343	p-V [1, 2]
**Who provided you with the main information about HPV vaccination?**			
Pediatricians/gynecologists	136 (9%)	52 (15%)	**<0.001**
Other healthcare workers	435 (29%)	129 (38%)	
Other	306 (21%)	77 (22%)	
No information received	601 (41%)	85 (25%)	

Results are expressed as mean (standard deviation, SD), or frequency (percentage).
[1]Fisher's exact test or Pearson's Chi-squared test for categorical variables
[2] The Student's t test confirmed by Mann-Whitney U test for continuous variables.
